# Prognostic impact of resistance to bortezomib and/or lenalidomide in carfilzomib‐based therapies for relapsed/refractory multiple myeloma: The Kyoto Clinical Hematology Study Group, multicenter, pilot, prospective, observational study in Asian patients

**DOI:** 10.1002/cnr2.1476

**Published:** 2021-06-14

**Authors:** Yuka Kawaji‐Kanayama, Tsutomu Kobayashi, Ayako Muramatsu, Hitoji Uchiyama, Nana Sasaki, Nobuhiko Uoshima, Mitsushige Nakao, Ryoichi Takahashi, Kazuho Shimura, Hiroto Kaneko, Miki Kiyota, Katsuya Wada, Yoshiaki Chinen, Koichi Hirakawa, Shin‐ichi Fuchida, Chihiro Shimazaki, Yayoi Matsumura‐Kimoto, Shinsuke Mizutani, Taku Tsukamoto, Yuji Shimura, Shigeo Horiike, Masafumi Taniwaki, Junya Kuroda, Kyoto Clinical Hematology Study Group (KOTOSG) Investigators

**Affiliations:** ^1^ Division of Hematology and Oncology, Department of Medicine Kyoto Prefectural University of Medicine Kyoto Japan; ^2^ Department of Hematology Japanese Red Cross Kyoto Daiichi Hospital Kyoto Japan; ^3^ Department of Hematology Japanese Red Cross Kyoto Daini Hospital Kyoto Japan; ^4^ Department of Internal Medicine Otsu Municipal Hospital Otsu Japan; ^5^ Department of Hematology Omihachiman Community Medical Center Omihachiman Japan; ^6^ Department of Hematology Aiseikai Yamashina Hospital Kyoto Japan; ^7^ Department of Hematology Matsushita Memorial Hospital Moriguchi Japan; ^8^ Department of Hematology Fukuchiyama City Hospital Fukuchiyama Japan; ^9^ Department of Hematology, Japan Community Health care Organization Kyoto Kuramaguchi Medical Center Kyoto Japan; ^10^ Center for Molecular Diagnostic and Therapeutics Kyoto Prefectural University of Medicine Kyoto Japan

**Keywords:** bortezomib, carfilzomib, lenalidomide, multiple myeloma

## Abstract

**Background:**

Combinatory strategies with carfilzomib (CFZ), a second‐generation proteasome inhibitor, plus dexamethasone (DEX) with or without lenalidomide (LEN) have shown promising efficacy for patients with relapsed/refractory multiple myeloma (RRMM) in pivotal clinical trials. However, their effects on patients who were resistance to bortezomib (BTZ) and/or LEN have not been fully evaluated in a daily practice setting.

**Aims:**

To evaluate the real‐world efficacy and safety of CFZ‐based treatments; that is, CFZ with LEN plus DEX (KRD therapy) and CFZ with DEX (KD therapy), in Asian patients, we conducted a multicenter pilot prospective observational study in the Kyoto Clinical Hematology Study Group.

**Methods and Results:**

All 50 patients with RRMM enrolled in this study were treated with CFZ‐based treatments between 2017 and 2019. KRD and KD were administered to 31 and 19 patients, respectively. The overall response rates (ORRs) were 80.6% with KRD and 73.7% with KD. Two‐year progression‐free survival (PFS) and overall survival (OS) were 58.5% and 79.7% with KRD, and 23.1% and 52.6% with KD. By multivariate analysis, refractoriness to BTZ and to LEN were identified as independent unfavorable factors for both PFS and OS. The common non‐hematologic AEs included hypertension (42.0%), fever (24.0%), fatigue (24.0%), and infection (16.0%). No serious heart failure was observed. This study is registered as UMIN000025108.

**Conclusion:**

This study suggests the need of the development of novel CFZ‐containing strategy which can overcome the refractoriness to BTZ and/or LEN, while both KRD and KD were shown to be mostly feasible in Asian patients in a daily practice setting.

## 1 INTRODUCTION

Multiple myeloma (MM) is the second most common hematologic malignancy,^1^ and the number of patients with MM has increased along with the increase in the elderly population. Despite the progress of therapeutic strategies through introduction of new drugs, such as proteasome inhibitors (PIs), immunomodulatory drugs (IMiDs) and monoclonal antibodies (MoAbs), in the past two decades, MM is still an incurable disease.^2^, ^3^ Relapse and acquisition of therapeutic resistance are inevitable in the clinical course of most patients with MM; therefore, achievement of long‐term survival with a favorable quality of life requires a therapeutic sequence tailored to each patient based on factors including myeloma‐related organ impairment, cytogenetic risk, fitness, comorbidities and treatment history.

Carfilzomib (CFZ) is a second‐generation, epoxyketone PI. By binding selectively and irreversibly to the β5 and β5i subunits and inhibiting chymotrypsin‐like constitutive proteasome and immunoproteasome activities, CFZ potently induces myeloma cell death, including cells with acquired resistance to bortezomib (BTZ), a first‐generation PI, in experimental settings.^4^, ^5^ Promising clinical efficacies of CFZ‐based therapies have also been reported in relapsed/refractory multiple myeloma (RRMM) in several pivotal clinical trials. In the ASPIRE trial, combinatory treatment of twice weekly CFZ with lenalidomide (LEN) and dexamethasone (DEX) (KRD therapy) was more efficacious than LEN and DEX only (Rd therapy) based on longer progression‐free survival (PFS) and overall survival (OS) in patients with RRMM.^6^, ^7^ In the ENDEAVOR trial, the superior efficacy of combination therapy of twice weekly CFZ and DEX (KD therapy) versus a combination of BTZ and DEX was also shown in RRMM, even in patients previously exposed to BTZ.^6^, ^7^, ^8^, ^9^, ^10^ However, patients refractory to BTZ and/or LEN were excluded in the ASPIRE trial, while those refractory to BTZ were excluded in the ENDEAVOR trial. Therefore, the efficacies of CFZ‐based therapy for RRMM in patients refractory to BTZ and/or LEN remain uncertain.

Information on the prognostic impact of prior resistance to BTZ and/or LEN in KRD therapy is also limited in real‐world observational studies,^11^, ^12^, ^13^ while such information is mostly lacking for KD therapy. Because the combination therapies containing BTZ and/or LEN are the standard of care for newly diagnosed MM, the failure of the first‐line treatment strongly associates with the resistance to BTZ and/or LEN. Therefore, therapeutic strategies those can overcome the resistance to BTZ and/or LEN are critically important as the salvage treatment for RRMM. Given that prior therapeutic resistance to BTZ and/or LEN may influence the effect of novel CFZ‐based therapy, such as KD plus the anti‐CD38 MoAb daratumumab,^14^ it is important to examine the prognostic significance of such prior resistance in KRD and KD therapies for the future development of new CFZ‐containing strategies for RRMM with the resistance to BTZ and/or LEN. In addition, there is little real‐world information on the efficacy and safety of CFZ‐based regimens in Asian patients with RRMM. Previous studies suggest differences in the adverse event (AE) profile of PIs between Caucasian and Asian patients. Several retrospective observational studies in Japanese patients with MM have shown a relatively high frequency of BTZ‐induced peripheral neuropathy (PN), compared to data for Caucasian patients, but there has been no direct comparative study.^15^, ^16^, ^17^ CFZ has also been suggested to cause severe cardiovascular AEs (CVAEs) in global clinical trials.^6^, ^7^, ^8^, ^9^, ^10^, ^11^, ^12^, ^13^, ^14^ Thus, there is a need to investigate the AE profile of CFZ‐based treatment in Asian patients in a daily practice setting.

Given these issues, we conducted a pilot prospective multicenter observational study, here designated as the KOTO‐CFZ study, to investigate the real‐world efficacy, factors related to treatment outcome, including the prior treatment status, and safety profiles of KD and KRD therapies in daily clinical practice for Asian patients with RRMM treated in centers belonging to the Kyoto Clinical Hematology Study Group (KOTOSG).

## 2 PATIENTS AND METHODS

### 2.1 Study design

Fifty patients with RRMM aged >20 years old who were scheduled to receive CFZ‐based therapies (i.e., KRD or KD) at nine centers in the KOTOSG were registered in the study before initiation of treatment from June 2017 to August 2019. The sample size of this study was determined based on the rules of pilot test.^18^, ^19^, ^20^ The data cut‐off date was March 31, 2021. No randomization was performed for selection of the treatment strategy for each patient, and the treatment modality was selected as KRD or KD at the discretion of a physician. KRD and KD were basically administered following the protocols established in pivotal trials, with dose modification due to AEs and/or a patient's condition permitted at the physician's discretion. Prophylaxis with acyclovir and sulfamethoxazole trimethoprim was recommended. The use of granulocyte‐colony stimulating factor (G‐CSF) for neutropenia was permitted.

Data were collected using a case report form for patient background factors, including age, gender, Eastern Cooperative Oncology Group (ECOG) performance status (PS), International Myeloma Working Group (IMWG) frailty score^21^ and comorbidities; serological laboratory data; disease information, such as the International Staging System (ISS) disease stage,^22^ cytogenetics, and paraprotein type; treatment history, including prior treatment types and responses; and treatment outcome with CFZ‐based therapy, including response, survival and AEs. The refractoriness to BTZ or LEN was defined as no objective response with therapy containing BTZ and/or LEN, and documented progression within 6 months after the last dose of BTZ or LEN. Regular serological and urinary tests during treatment were prespecified in the study protocol, and additional examinations were allowed as needed. An electrocardiogram and echocardiogram were obtained before the start of CFZ‐based treatment, at the end of the third course of treatment, and at the end of treatment with CFZ.

The study was conducted in compliance with the Guidelines for Good Clinical Practice and the Declaration of Helsinki, and the study protocol was approved by local institutional review boards. All patients provided written informed consent at the study enrollment before the initiation of CFZ‐based treatment. This study is registered as UMIN000025108.

### 2.2 Study endpoint

The primary endpoint was overall response rate (ORR). Treatment response was assessed using the IMWG criteria^23^ and ORR was defined as the sum of rates of stringent complete response (sCR), CR, very good partial response (VGPR) and PR. The secondary endpoints were PFS, OS, and safety. PFS was defined as the time from the date of treatment initiation to progression or death, and OS as the time from the date of treatment initiation to death. PFS was censored at the end of CFZ‐based treatment in patients who proceeded to a planned next treatment. AEs were graded using the National Cancer Institute Common Terminology Criteria for Adverse Events (CTCAE) ver. 4.0. Regarding hypertension, grade 2 or higher was defined as clinically significant.

### 2.3 Statistical analysis

A Fisher exact test was used to compare categorical variables and a Mann–Whitney‐U test was used to compare continuous variables between two groups. The Kaplan–Meier method was used for survival analysis, with a log‐rank test for comparison of the survival curves. Prognostic factors were identified using Cox proportional hazards model analysis. The confidence interval (CI) was 95% and *p* < .05 was considered to be significant. All statistical analyses were performed with EZR ver. 1.37.^24^

## 3 RESULTS

### 3.1 Background of patients

The characteristics of the 50 patients are summarized in Table 1. All patients were Asian. Thirty‐one patients received KRD therapy and 19 received KD therapy. The median ages were 67 years (range, 41–81) in all 50 patients, 67 years in the KRD group, and 70 years in the KD group. Two patients were classified as ECOG‐PS 3; 10 (20.0%) were frail based on the IMWG frailty score; and 17 (34.0%) were in ISS stage III. The median number of prior regimens was 2 (range, 1–7); the rates of prior exposure to BTZ and LEN were 90.0% (45/50) and 70.0% (35/50), respectively; and there were 21 (42.0%) and 19 (38.0%) BTZ‐ and LEN‐refractory patients, respectively. The rates of histories of hypertension, diabetes mellitus and hyperlipidemia were 30.0%, 20.0%, and 18.8%, respectively. There was no significant difference in patient backgrounds between the KRD and KD groups.

**TABLE 1:** Patient background

Item	Total (*n* = 50)	KRD (*n* = 31)	KD (*n* = 19)	*p*
Age, median (range)	67 (41–81)	67 (47–81)	70 (41–81)	.063
Gender, male/female	24/26	15/16	9/10	1
ECOG‐PS, 0/1/2/3/4/NA	24/16/4/4/2/0	15/11/3/2/0/0	9/5/1/2/2/0	.503
Frailty score fit/intermediate/frail/NA	35/5/10/0	24/3/4/0	11/2/6/0	.246
M‐protein				
IgG/IgA/BJP/others	32/9/8/1	21/4/5/1	11/5/3/0	.670
κ/λ	29/21	20/11	9/10	.255
ISS, I/II/III/NA^a^	14/17/17/1	9/10/10/1	5/7/7/0	1
Durie and Salmon^a^				
1/2/3/NA	5/8/36/1	4/4/22/1	1/4/14/0	.636
A/B/NA	39/7/4	23/4/4	16/3/0	1
Number of prior regimens, median (range)	2 (1–7)	1 (1–4)	2 (1–7)	.067
Prior BTZ exposure, *n* (%)	45 (90.0)	28 (90.3)	17 (89.5)	.636
Prior LEN exposure, *n* (%)	35 (70.0)	22 (71.0)	13 (68.4)	1
Refractory to BTZ, *n* (%)	21 (42.0)	12 (38.7)	9 (47.4)	.570
Refractory to LEN, *n* (%)	19 (38.0)	10 (32.3)	9 (47.4)	.372
Past medical history, *n* (%)				
Hypertension	15 (30.0)	7 (22.6)	8 (42.1)	.210
Diabetes mellitus	10 (20.0)	6 (19.4)	4 (21.1)	1
Hyperlipidemia	9 (18.0)	6 (19.4)	3 (15.8)	1
Peripheral neuropathy	3 (6.0)	2 (6.5)	1 (5.3)	1
Pulmonary complications	3 (6.0)	2 (6.5)	1 (5.3)	1
Cerebrovascular events	1 (2.0)	0	1 (5.3)	.380
Angina pectoris	1 (2.0)	0	1 (5.3)	.380
Creatinine ≥2.0 mg/dl, *n* (%)	5 (10.0)	1 (3.2)	4 (21.1)	.062
High‐risk cytogenetics by FISH, *n* (%) t(4;14) or t(14;16)	13 (26.0)	7 (22.6)	6 (31.6)	.521

Abbreviations: BJP, Bence‐Jones protein type; BTZ, bortezomib; ISS, International Staging System; FISH, fluorescence in situ hybridization; KD, carfilzomib and dexamethasone; KRD, carfilzomib, lenalidomide and dexamethasone; PS, performance status; NA, not available.

^a^ Staging at diagnosis.

### 3.2 Treatment outcome and response

With a median follow‐up of 28.3 months, the median numbers of treatment cycles in the KRD and KD groups were 4 (range, 1–41) and 4 (range, 1–25), respectively. Median single dose of CFZ was 27 mg/m^2^ (range 20–27) in KRD group and 56 mg/m^2^ (range 20–56) in KD group, and median single dose of DEX was 20 mg/body (KRD; range 8–40, KD; range 4–40) in both groups (Table S1). All patients received a twice‐weekly CFZ regimen. Of all 50 patients, 2 (4.0%) were still under treatment with CFZ‐based therapy at the data cut‐off, while 48 had discontinued therapy due to AEs (*n* = 7, 14.0%), including CVAEs (*n* = 3); disease progression (*n* = 18, 36.0%); insufficient response that resulted in a treatment change (*n* = 5, 10.0%); a pre‐planned treatment change (*n* = 13, 26.0%), including proceeding to high‐dose chemotherapy with autologous stem cell transplantation (HDT/ASCT), typically after four cycles of KRD or KD (*n* = 9), and proceeding to LEN maintenance after KRD (*n* = 4); and other reasons (*n* = 5, 10.0%) (Table S1). In this cohort, we performed HDT/ASCT after CFZ‐based treatments in patients who were fit and under 70 years old.

**FIGURE 1:**
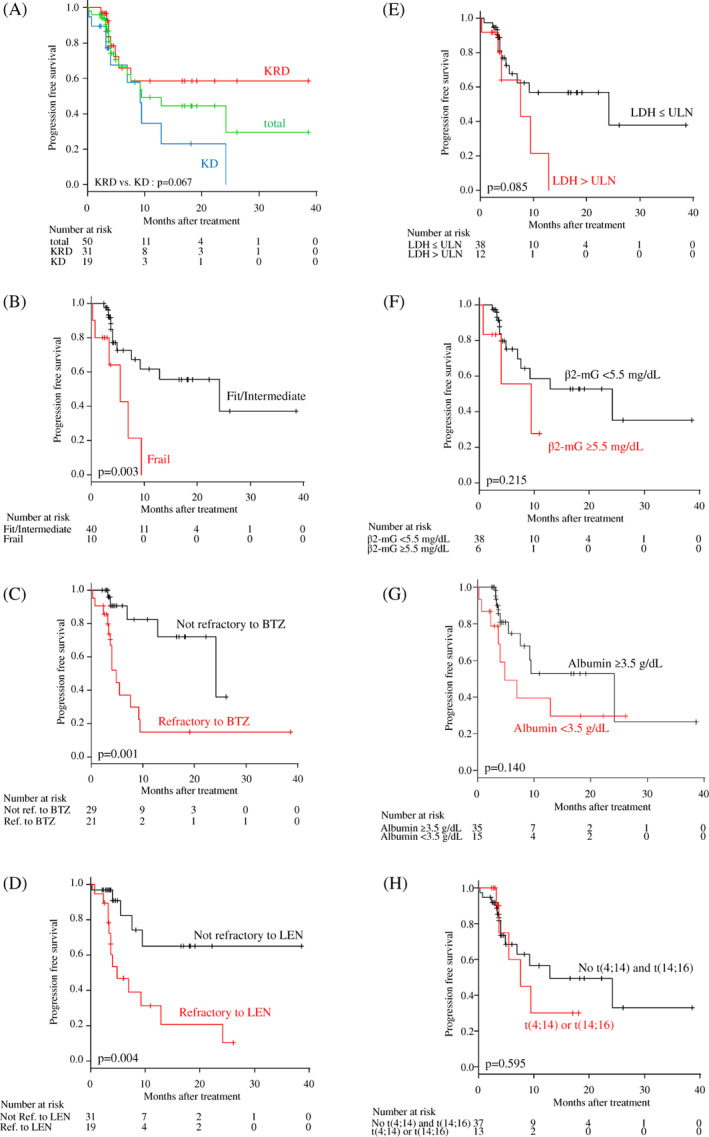
Kaplan–Meier curves for progression‐free survival (PFS) with carfilzomib‐based treatments. PFS according to (A) treatment type, (B) patient condition, (C) refractoriness to bortezomib (BTZ), (D) refractoriness to lenalidomide (LEN), (E) serum lactate dehydrogenase (LDH) level, (F) serum β2‐microglobulin (mG) level, (G) serum albumin level, and (H) high‐risk cytogenetics (t(4;14) or t(14;16)). ref., refractory; ULN, upper limit of normal

In 50 patients evaluable for best response, ORR was 78.0% (*n* = 39), including 11 CR/sCR (22.0%), 10 VGPR (20.0%), and 18 PR (36.0%). ORR was 80.6% (51.6% CR/sCR and VGPR) and 73.7% (26.3% CR/sCR and VGPR) in the KRD and KD groups, respectively. There was no significant difference in the rate of ORR between in the KRD group and KD group (*p* = .475) (Table S1). The two‐year PFS and OS of all patients were 44.4% (95% CI: 24.9–62.2) and 69.5% (95% CI: 54.5–80.4), respectively. The median PFS was 9.4 months, and the median OS was not reached. According to Kaplan–Meier analysis, KRD group had significantly longer OS than KD group (median, not reached vs. 28.1 months, *p* = .001), while there was no difference in PFS between two treatment groups (median, not reached vs. 9.3 months, *p* = .067) (Figure 1A and Figure S1A).

### 3.3 Factors associated with survival

Next, we analyzed the prognostic impact of frailty defined by the IMWG frailty score, number of prior regimens (≥2), refractoriness to BTZ or LEN, serum lactate dehydrogenase (LDH) level (> upper limit of normal [ULN] or ≤ULN), serum β2‐microgloblin (β2‐mG) level (≥5.5 mg/dl), serum albumin level (<3.5 g/dl), and high‐risk cytogenetics (t(4;14) or t(14;16)) on PFS and OS. Univariate analyses suggested the prognostic impacts of frailty and refractoriness to BTZ and/or LEN on PFS (Figure 1B–H); and of frailty, refractoriness to BTZ, serum LDH level, serum β2‐mG level, and serum albumin level on OS (Figure S1B–H). Subsequently, we performed multivariate analyses with these six factors associated with PFS or OS in univariate analysis. As the results, the refractoriness to BTZ and the refractoriness to LEN were identified as independent unfavorable risk factors for PFS; and the refractoriness to BTZ, the refractoriness to LEN, and serum β2‐mG level were identified as independent unfavorable risk factors for OS (Table 2).

**TABLE 2:** Univariate and multivariate analyses for PFS and OS

	PFS				OS			
	Univariate analysis		Multivariate analysis		Univariate analysis		Multivariate analysis	
	HR (95%CI)	*p*	HR (95%CI)	*p*	HR (95%CI)	*p*	HR (95%CI)	*p*
IMWG frailty score, frail	4.191 (1.504–11.680)	.006	‐	‐	3.536 (1.268–9.860)	.016	‐	‐
Refractory to BTZ, yes	5.025 (1.778–14.200)	.002	6.402 (1.880–21.800)	.003	4.330 (1.610–11.650)	.004	3.227 (1.074–9.700)	.037
Refractory to LEN, yes	4.135 (1.465–11.670)	.007	8.751 (2.273–33.690)	.002	2.447 (0.964–6.214)	.060	3.376 (1.159–9.829)	.026
Serum LDH level, >ULN	2.213 (0.606–8.087)	.230	‐	‐	4.328 (1.608–11.650)	.004	‐	‐
Serum β2‐mG level, ≥5.5 mg/dl	1.326 (0.466–3.771)	.597	‐	‐	6.035 (1.946–18.720)	.002	4.949 (1.520–16.110)	.008
Serum albumin level, < 3.5 g/dl	1.988 (0.718–5.508)	.149	‐	‐	2.993 (1.186–7.556)	.020	‐	‐

Abbreviations: BTZ, bortezomib; IMWG, international myeloma working group; LDH, lactate dehydrogenase; LEN, lenalidomide; ULN, upper limit of normal, β2‐mG, β2‐microglobulin.

**FIGURE 2:**
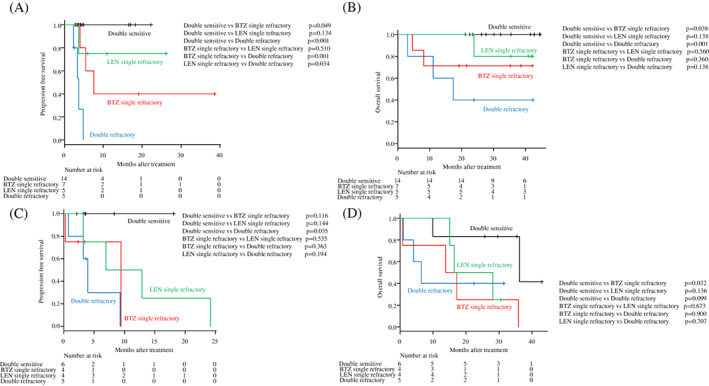
Kaplan–Meier curves for progression‐free survival (PFS) and overall survival (OS) for KRD (A; PFS, B; OS) and KD (C; PFS, D; OS) therapy, according to refractoriness to BTZ and/or LEN

The relationship between resistance to BTZ and/or LEN and survival with CFZ‐based therapy was next examined in more detail. The patients were divided into four groups: those sensitive to both BTZ and LEN (double‐sensitive), refractory to BTZ but not to LEN, refractory to LEN but not to BTZ, and refractory to both BTZ and LEN (double‐refractory). Survival outcomes with KRD or KD therapy were compared among the groups. Despite the small number of patients in each group, patients who were double‐refractory to BTZ and LEN either significantly had or tended to have a poor prognosis compared with those with single resistance to BTZ or LEN; and the double‐sensitive to BTZ and LEN may be a favorable predictor for PFS and OS in both KRD and KD therapy (Figure 2). In addition, the presence of poor cytogenetic abnormalities, that is, t(4;14) or t(14;16), did not significantly affect PFS and OS in our cohort (Figure 1H and Figure S1H), while the prognostic impact of del(17p) or 1q abnormality was not evaluable due to missing data.

### 3.4 Safety and adverse events

No grade 5 AEs occurred in the study. Most AEs occurred within the first treatment course (hematological AEs: median 1 [range 1–14]; non‐hematological AEs: median 1 [range 1–8]). Hematological AEs occurred in 43 patients (86.0%), including anemia (64.0%), lymphopenia (60.0%), thrombocytopenia (50.0%) and neutropenia (46.0%), and approximately half of hematologic AEs were grade 3 to 4. No febrile neutropenic episodes occurred in our cohort. CVAEs included hypertension in 21 patients (42.0%), arrhythmia in 7 (14.0%), dyspnea in 2 (4.0%), pulmonary edema in 1 (2.0%), and congestive heart failure in 1 (2.0%). Hypertension (HTN) was defined as event which manifested or deteriorated after CFZ‐based treatment, and HTN, which required medical intervention, was defined as a grade 2 event. No ischemic heart disease or cardiomyopathy was detected. PN occurred in 3 patients (6.0%) who all had PN at baseline. There were significantly higher incidences of grade 3 to 4 HTN, all‐grade nausea, and all‐grade delirium in the KD group compared to the KRD group, but no difference in the frequency of other AEs between the two regimens (Table 3).

**TABLE 3:** Adverse events

	Total (*n* = 50)		KRD (*n* = 31)		KD (*n* = 19)		*p*	
	All	G3‐4	All	G3‐4	All	G3‐4	All	G3‐4
**Hematological adverse events, *n* (%)**								
Anemia	32 (64.0)	10 (20.0)	20 (64.5)	5 (16.1)	12 (63.2)	5 (26.3)	1	.474
Lymphopenia	30 (60.0)	21 (42.0)	18 (58.1)	10 (32.3)	12 (63.2)	10 (52.6)	.774	.255
Thrombocytopenia	25 (50.0)	13 (26.0)	13 (41.9)	9 (29.0)	12 (63.2)	5 (26.3)	.255	1
Neutropenia	23 (46.0)	12 (24.0)	12 (38.7)	8 (25.8)	11 (57.9)	4 (21.1)	.383	1
**Non‐hematological adverse events, *n* (%)**								
Hypertension	21 (42.0)	3 (6.0)	11 (35.5)	0	10 (52.6)	3 (15.8)	.255	.049
Fever	12 (24.0)	0	7 (22.6)	0	5 (26.3)	0	1	‐
Fatigue	12 (24.0)	2 (4.0)	6 (19.4)	1 (3.2)	6 (31.6)	1 (5.3)	.496	1
Infection	8 (16.0)	3 (6.0)	4 (12.9)	1 (3.2)	4 (21.1)	2 (10.5)	.459	.549
Skin rash	7 (14.0)	1 (2.0)	5 (16.1)	1 (3.2)	2 (10.5)	0	.229	1
Arrhythmia	7 (14.0)	0	3 (9.7)	0	4 (21.1)	0	.404	‐
AST/ALT* increased	5 (10.0)	3 (6.0)	2 (6.5)	1 (3.2)	3 (15.8)	2 (10.5)	.355	.549
Constipation	5 (10.0)	0	3 (9.7)	0	2 (10.5)	0	1	‐
QTc interval prolonged	5 (10.0)	0	4 (12.9)	0	1 (5.3)	0	.637	‐
Anorexia	4 (8.0)	1 (2.0)	1 (3.2)	0	3 (15.8)	1 (5.3)	.147	.380
Diarrhea	4 (8.0)	0	2 (6.5)	0	2 (10.5)	0	.618	‐
Thrombosis	4 (4.0)	1 (2.0)	3 (9.7)	1 (3.2)	1 (5.3)	0	.255	1
Hypoxia	3 (6.0)	3 (6.0)	1 (3.2)	1 (3.2)	2 (10.5)	2 (10.5)	.549	.549
Nausea	3 (6.0)	0	0	0	3 (15.8)	0	.049	‐
Edema	3 (6.0)	0	1 (3.2)	0	2 (10.5)	0	.549	‐
Peripheral neuropathy	3 (6.0)	0	2 (6.5)	0	1 (5.3)	0	1	‐
Delirium	3 (6.0)	2 (10.5)	0	0	3 (15.8)	2 (10.5)	.049	.140
Dyspnea	2 (4.0)	2 (4.0)	0	0	2 (10.5)	2 (10.5)	.140	.140
Pulmonary edema	1 (2.0)	1 (2.0)	0	0	1 (5.3)	1 (5.3)	1	1
Heart failure	1 (2.0)	0	1 (3.2)	0	0	0	.380	‐
ST segment elevation	1 (2.0)	0	1 (3.2)	0	0	0	.380	‐

### 3.5 Discussion

In our real‐world cohort of 50 Asian patients with RRMM registered in this KOTO‐CFZ study, ORR and median PFS were 80.6% and not reached with KRD therapy and 73.7% and 9.3 months with KD therapy with a median follow‐up time of 28.3 months. These ORRs are comparable to those reported in the ASPIRE trial for KRD^6^ and ENDEAVOR trial for KD,^8^ but Kaplan–Meier curve for PFS with KRD and KD in our cohort seemed to be inferior to median PFS of 26.3 months in the APIRE trial (median follow‐up: 32.3 months) and to median PFS of 17.0 months in the ENDEAVOR trial (median follow‐up: 11.9 months). However, care is required in comparing results among clinical studies, and there are several possible reasons for what may be meaningful differences.

One conceivable reason for the inferior survival period compared with those in the ASPIRE and ENDEAVOR trials may be the higher proportions of patients with prior treatment histories with BTZ (90.0%), LEN (70.0%) and BTZ and LEN (62.0%) in our cohort. The rates of prior treatment histories with BTZ, LEN, and BTZ and LEN were 66%, 59% and 37%, respectively, in patients treated with KRD in the ASPIRE trial; and 54%, 70% and 34%, respectively, in patients treated with KD in the ENDEAVOR trial.^6^, ^7^, ^8^ Perhaps more importantly, our cohort included 42.0% of BTZ‐refractory and 38.0% of LEN‐refractory cases, whereas patients who progressed during BTZ‐containing treatment and LEN‐refractory patients were excluded in the ASPIRE trial, and PI‐refractory patients were excluded in the ENDEAVOR trial.^6^, ^7^, ^8^

Daratumumab in combination with BTZ or LEN, or combinatory/sequential use of BTZ and LEN are the current standard of care as the first‐ to second‐line treatment for newly diagnosed MM.^25^, ^26^, ^27^, ^28^, ^29^, ^30^, ^31^ Thus, CFZ‐based treatment is often utilized as a salvage strategy for patients with acquired resistance to BTZ and/or LEN. Therefore, our patient population including patients refractory to BTZ and/or LEN more faithfully mirrors the current situation in daily clinical practice settings for RRMM, and the lack of data on patients resistant to BTZ and/or LEN is a critical data gap in the ASPIRE and ENDEAVOR trials.^6^, ^7^ Importantly, our study showed that refractoriness to BTZ and/or LEN was significantly associated with shorter PFS and OS. In particular, the prognosis was poorer in patients who were double‐refractory to BTZ and LEN, compared with other statuses. Several basic studies have shown cross‐resistance among PIs,^32^, ^33^ despite the more powerful anti‐myeloma effect of CFZ that may potentially overcome resistance to BTZ.^4^ In addition, several early phase clinical trials have shown an unsatisfactory response to CFZ in BTZ‐refractory patients, especially in those with RRMM.^34^ The efficacy in the current study may reflect the findings in these basic and clinical studies, and suggests the importance of insight into drug sensitivity based on prior treatment in selection of CFZ‐based therapies compared to other approaches. For instance, it has been reported that addition of daratumumab to KD may improve the outcomes of patients with acquired resistance to LEN.^14^ In addition, patients in KRD group showed significantly longer OS than in KD group, and KRD also provided trend for long PFS compared with KD, although not significantly, in this study. These trends seemed to be consistent with previously reported data.^35^

With regard to AEs, CFZ has been shown to have less off‐target activity against enzymes other than those in the proteasome and immunoproteasome,^36^ and this may be associated with the different toxicity profile of CTZ compared with BTZ; for example, the low incidence of PN.^6^, ^7^, ^8^, ^9^, ^10^, ^11^, ^12^, ^13^, ^14^ Hematological AEs were most common in our cohort, and there was no significant difference in the frequencies of hematologic AEs with KRD and KD therapy. Regarding non‐hematologic AEs, a high frequency of CVAEs has been a critical concern in use of CFZ for myeloma.^6^, ^7^, ^8^, ^9^, ^10^, ^11^, ^12^, ^13^ HTN was the most frequent non‐hematological AE in our cohort, and HTN over grade 2 occurred more frequently in our patients compared with previous reports.^6^, ^7^, ^8^, ^9^, ^10^, ^11^, ^12^, ^13^, ^37^, ^38^, ^39^, ^40^ Arrythmia occurred in 7 patients (14.0%), with all events being grade 1–2, detected on a regular electrocardiogram without symptoms, and requiring no medication. Somewhat surprisingly, only one patient had grade 1 heart failure, giving a much lower rate than that in a previous study of CFZ in an Asian cohort.^41^

These results for AEs might be biased by patient selection in our cohort, since no patient had a prior history of severe cardiovascular disease and only one had angina pectoris, while 30.0% of the patients had a history of HTN. It is possible that CFZ‐based therapy was avoided in patients with high cardiovascular risks in our study group. However, this kind of patient selection based on clinical judgment may eliminate an opportunity for CFZ therapy in patients in whom CFZ would actually be safe. Risk factors for CVAEs with CFZ remain uncertain, since one meta‐analysis suggested an association of a higher dosage of CFZ with CVAEs,^41^ while another linked concurrent use of IMiD, but not the CFZ dosage, and CVAEs.^42^

In our cohort, whereas CFZ single dosage did not associate with the frequency of all grade HTN in this study, KD therapy was significantly associated with a higher incidence of grade 3–4 HTN, compared with KRD therapy. At a glance, this suggests a need for caution regarding HTN in use of higher dosage CFZ; however, there were very few events of grade 3–4 HTN and this finding requires confirmation. We are currently conducting additional studies to identify clinical presentations and biomarkers for prediction of CVAEs with CFZ. Fever and fatigue were the second commonest non‐hematologic AEs, with occurrence in >20% of patients. In contrast, diarrhea occurred in only 8.0% of our patients, despite being the most frequent non‐hematological AE in the ASPIRE and ENDEAVOR trials.^6^, ^7^, ^8^, ^9^ Three patients with BTZ‐induced PN at baseline complained of persistent PN after enrollment; however, CFZ did not exacerbate PN. All AEs were manageable in actual clinical practice. Therefore, CFZ‐based treatment was shown to be mostly feasible, even in elderly or frail patients, and these results collectively support the idea that CFZ‐based therapy should not be restricted by a frail status alone.^43^

This study has several key limitations. First, it was performed as a non‐interventional observational study; therefore, the detailed treatment plans for the CFZ‐based therapies were not defined and were not uniform among patients. However, the reason for this study design was to investigate the actual condition in daily practice with use of CFZ for RRMM in community hospitals. Second, the sample size of this study was small due to a pilot study nature which might potentially affect the statistical power. Third, our data lack sufficient information of high‐risk cytogenetics, especially del(17p) and 1q gain. This is also related to the small sample size, which made it impossible to investigate the prognostic impact of high‐risk cytogenetics in our series. Nevertheless, our results show that CFZ‐containing therapies can be used safely in a real‐world setting in elderly Asian patients with RRMM with a wide variety of backgrounds, treatment histories, and disease status.

In conclusion, this study shows that CFZ‐based treatment is effective and mostly feasible in Asian patients with RRMM in a real‐world clinical setting, however, a prior history of resistance to BTZ and/or LEN may impair for the efficacy and the outcome of CFZ‐based treatment. This suggests the need of the development of novel CFZ‐containing strategy which can overcome the refractoriness to BTZ and/or LEN by, for instance, adding upcoming new agents, such as a monoclonal antibody, an antibody‐drug conjugate, selinexor or venetoclax, and also the need of the next prospective study for evaluating efficacies of those strategies with larger sample size.

## CONFLICT OF INTEREST

This study is supported by Ono Pharmaceutical. J.K. has received research funding from Bristol‐Myers Squibb, Sysmex, Celgene, Ono Pharmaceutical, Otsuka Pharmaceutical, Sanofi, Kyowa Kirin, Chugai Pharmaceutical, Eisai, Astellas Pharma, Dainippon Sumitomo Pharma, Nippon Shinyaku, Takeda, Shionogi, Asahi Kasei, Daiichi Sankyo, MSD, Taiho Pharmaceutical, Fujimoto Pharmaceutical and Abbvie; has received honoraria from Bristol‐Myers Squibb, Janssen Pharmaceutical K.K, Celgene Corporation, Ono Pharmaceutical, Takeda, Sanofi, Kyowa Kirin, Chugai Pharmaceutical, Eisai, Astellas Pharma, Nippon Shinyaku, Dainippon Sumitomo Pharma, Daiichi Sankyo, Fujimoto Pharmaceutical, Abbvie and Otsuka Pharmaceutical; and is a consultant for Janssen Pharmaceutical K.K, Celgene, Bristol‐Myers Squibb, Sanofi and Abbvie. T.K. has received honoraria from Chugai Pharmaceutical, Ono Pharmaceutical, Eisai, and Nippon Shinyaku. T.T. has received research funding from Nippon Shinyaku. All other authors have no conflict of interest.

## AUTHOR CONTRIBUTIONS

All authors read and approved the final manuscript. *Analyzed and Interpreted the Data*, J.K., T.K. and Y.K.; *Study Conception and Design*, J.K. and T.K.; *Data Acquisition*, Y.K., A.M., H.U, N.S., N.U., M.N., R.T., K.S., H.K., M.K., K.W., Y.C., K.H., S.F., C.S., Y.M., S.M., T.T., Y.S., S.H., and M.T.; *Statistical Analysis*, T.K. and Y.K.; *Drafted the Manuscript*, Y.K.; *Revision of the Manuscript*, J.K. and T.K.

## ETHICS STATEMENT

This study was approved by the Institutional Review Board of Kyoto Prefectural University of Medicine and institutional review boards of all enrolled institutes and was conducted according to the principles of the Declaration of Helsinki. All patients provided written informed consent.

## Supporting information


**Table S1.** Outcomes and responses to CFZ‐based treatment.

**Figure S1.** Kaplan–Meier curves for overall survival (OS) with carfilzomib‐based therapy. OS according to (A) treatment type, (B) patient condition, (C) refractoriness to bortezomib (BTZ), (D) refractoriness to lenalidomide (LEN), (E) serum lactate dehydrogenase (LDH) level, (F) serum β2‐microglobulin (mG) level, (G) serum albumin level, and (H) high‐risk cytogenetics (t(4;14) or t(14;16)). ref., refractory; ULN, upper limit of normal.

## Data Availability

The authors confirm that the data supporting the findings of this study are available within the article and its supplementary materials (Figure S1; Table S1).
