# Supplementary material for: Prognostic impact of resistance to bortezomib and/or lenalidomide in carfilzomib‐based therapies for relapsed/refractory multiple myeloma: The Kyoto Clinical Hematology Study Group, multicenter, pilot, prospective, observational study in Asian patients
Source: Cancer Rep (Hoboken). 2021 Jun 14;5(2):e1476. doi: 10.1002/cnr2.1476 (PMC8842705; doi:10.1002/cnr2.1476)
Supplement: Supplementary file 1 — Table S1. Outcomes and responses to CFZ‐based treatment. [file CNR2-5-e1476-s002.docx]

**Table S1. Outcomes and responses to CFZ-based treatment**

| Treatment outcome, n (%) | Total (n=50) | KRD (n=31) | KD (n=19) |  |
| --- | --- | --- | --- | --- |
| Treatment discontinuation  Cause of discontinuation  PD  Insufficient response  Adverse events  Fatigue  Dyspnea  Premature atrial contraction  Thrombosis  Skin rash  Elevation of BNP  Pre-planned treatment switch  To HDT/ASCT  To LEN maintenance  Patient request  Treatment-unrelated complication | 48 (98.0%)  18 (36.0%)  5 (10.0%)  7 (14.0%)  2  1  1  1  1  1  13 (26.0%)  9 (18.0%)  4 (8.0%)  4 (8.0%)  1 (2.0%) | 29 (93.5%)  8 (25.8%)  4 (12.9%)  3 (9.7%)  0  0  1  0  1  1  10 (32.3%)  6 (19.4%)  4 (12.9%)  3 (9.7%)  1 (3.2%) | 19 (100.0%)  10 (52.6%)  1 (5.3%)  4 (21.1%)  2  1  0  1  0  0  3 (15.8%)  3 (15.8%)  0 (0.0%)  1 (5.3%)  0 (0.0%) |  |
| Median dose | Total (n=50) | KRD (n=31) | KD (n=19) | *p* |
| CFZ, median (range) mg/m^2^ | - | 27 (20-27) | 56 (20-56) | - |
| LEN, median (range) mg/body | - | 15 (5-25) | - | - |
| DEX, median (range) mg/body | 20 (4-40) | 20 (8-40) | 20 (4-40) | 0.586 |
| Best response, n (%) | Total (n=50) | KRD (n=31) | KD (n=19) | *p* |
| CR/sCR | 11 (22.0) | 9 (29.0) | 2 (10.5) | 0.475 |
| VGPR | 10 (20.0) | 7 (22.6) | 3 (15.8) |  |
| PR | 18 (36.0) | 9 (29.0) | 9 (47.4) |  |
| SD | 5 (10.0) | 3 (9.7) | 2 (10.5) |  |
| PD | 6 (12.0) | 3 (9.7) | 3 (15.8) |  |
| ORR, % | 78.0 | 80.6 | 73.7 |  |

Abbreviations: CFZ, carfilzomib; LEN, lenalidomide; DEX, dexamethasone; CR, complete response; sCR, stringent CR; VGPR, very good partial response; PR, partial response; SD, stable disease; PD, progressive disease; ORR, overall response rate; BNP, brain natriuretic peptide; HDT/ASCT, high-dose chemotherapy with autologous stem cell transplantation.
