# Supplementary material for: Prognostic impact of resistance to bortezomib and/or lenalidomide in carfilzomib‐based therapies for relapsed/refractory multiple myeloma: The Kyoto Clinical Hematology Study Group, multicenter, pilot, prospective, observational study in Asian patients
Source: Cancer Rep (Hoboken). 2021 Jun 14;5(2):e1476. doi: 10.1002/cnr2.1476 (PMC8842705; doi:10.1002/cnr2.1476)
Supplement: Supplementary file 2 — Figure S1. Kaplan–Meier curves for overall survival (OS) with carfilzomib‐based therapy. OS according to (A) treatment type, (B) patient condition, (C) refractoriness to bortezomib (BTZ), (D) refractoriness to lenalidomide (LEN), (E) serum lactate dehydrogenase (LDH) level, (F) serum β2‐microglobulin (mG) level, (G) serum albumin level, and (H) high‐risk cytogenetics (t(4;14) or t(14;16)). ref., refractory; ULN, upper limit of normal. [file CNR2-5-e1476-s001.pptx]

## Slide 1
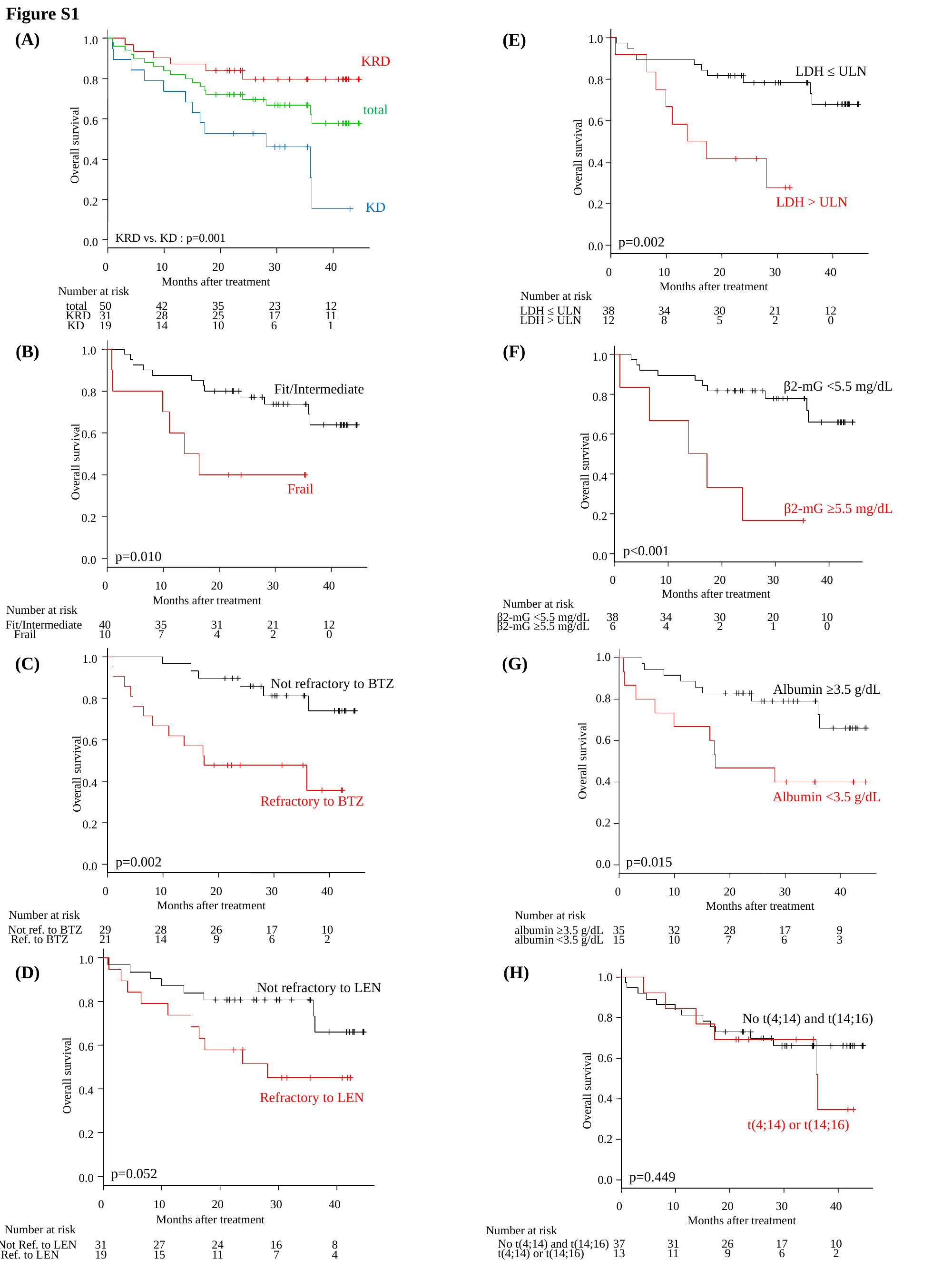

1.0
0.8
0.6
Overall survival
0.4
0.2
0.0
0
10
20
30
40
Months after treatment
Number at risk
LDH ≤ ULN
38
34
30
21
12
LDH > ULN
12
8
5
2
0
1.0
0.8
0.6
Overall survival
0.4
0.2
0.0
0
10
20
30
40
Months after treatment
Number at risk
total
50
42
35
23
12
KRD
31
28
25
17
11
KD
19
14
10
6
1
Figure S1
(A)
(E)
KRD
LDH ≤ ULN
total
LDH > ULN
KD
KRD vs. KD : p=0.001
p=0.002
1.0
0.8
0.6
Overall survival
0.4
0.2
0.0
0
10
20
30
40
Months after treatment
Number at risk
Fit/Intermediate
40
35
31
21
12
Frail
10
7
4
2
0
1.0
0.8
0.6
Overall survival
0.4
0.2
0.0
0
10
20
30
40
Months after treatment
Number at risk
β2-mG <5.5 mg/dL
38
34
30
20
10
β2-mG ≥5.5 mg/dL
6
4
2
1
0
(B)
(F)
β2-mG <5.5 mg/dL
Fit/Intermediate
Frail
β2-mG ≥5.5 mg/dL
p<0.001
p=0.010
1.0
0.8
0.6
Overall survival
0.4
0.2
0.0
0
10
20
30
40
Months after treatment
Number at risk
Not ref. to BTZ
29
28
26
17
10
Ref. to BTZ
21
14
9
6
2
1.0
0.8
0.6
Overall survival
0.4
0.2
0.0
0
10
20
30
40
Months after treatment
Number at risk
albumin ≥3.5 g/dL
35
32
28
17
9
albumin <3.5 g/dL
15
10
7
6
3
(C)
(G)
Not refractory to BTZ
Albumin ≥3.5 g/dL
Albumin <3.5 g/dL
Refractory to BTZ
p=0.015
p=0.002
1.0
0.8
0.6
Overall survival
0.4
0.2
0.0
0
10
20
30
40
Months after treatment
Number at risk
Not Ref. to LEN
31
27
24
16
8
Ref. to LEN
19
15
11
7
4
(D)
(H)
1.0
0.8
0.6
Overall survival
0.4
0.2
0.0
0
10
20
30
40
Months after treatment
Number at risk
No t(4;14) and t(14;16)
37
31
26
17
10
t(4;14) or t(14;16)
13
11
9
6
2
Not refractory to LEN
No t(4;14) and t(14;16)
Refractory to LEN
t(4;14) or t(14;16)
p=0.052
p=0.449
